# Accreditation of occupational health services in Norway

**DOI:** 10.1093/occmed/kqv120

**Published:** 2015-08-14

**Authors:** A. Lie, O. Bjørnstad

**Affiliations:** Secretariat for Occupational Health Services, National Institute of Occupational Health, N-0033 Oslo, Norway.

**Keywords:** Accreditation, Norway, occupational health service, quality, survey.

## Abstract

**Background:**

In 2010, an accreditation system for occupational health services (OHS) in Norway was implemented.

**Aims:**

To examine OHS experiences of the accreditation system in Norway 4 years after its implementation.

**Methods:**

A web-based questionnaire was sent to all accredited OHS asking about their experiences with the accreditation system. Responses were compared with a similar survey conducted in 2011.

**Results:**

The response rate was 76% (173/228). OHS reported that the most common changes they had had to make to achieve accreditation were: improvement of their quality assurance system (53%), a plan for competence development (44%) and increased staffing in occupational hygiene (36%) and occupational medicine (28%). The OHS attributed improved quality in their own OHS (56%) and in OHS in Norway (47%), to the accreditation process.

**Conclusions:**

The accreditation system was well accepted by OHS, who reported that it had improved the quality of their OHS and of OHS in Norway. The results are similar to the findings of a 2011 survey.

## Introduction

Norway has 5 million inhabitants and a workforce of 2.7 million. Employers of approximately half of the workforce are obliged to have occupational health services (OHS) because their employees are particularly vulnerable to disease or injury [1]. OHS who want to supply such businesses must be accredited by the Labour Inspection Authority [2]. The accreditation system was introduced in Norway in 2010.

Accreditation is conducted by the Labour Inspection Authority with no charge for the OHS. To become accredited, OHS must have a quality assurance system and at least three full time equivalents (FTEs) OHS professionals with expertise in occupational medicine, occupational hygiene, ergonomics, the psychosocial work environment and systematic health, safety and environmental work [2]. Accreditation lasts 5 years and the OHS will be visited by the accreditation unit at least once during this 5-year period.

During the preliminary work for a revised Work Environment Act, doubts were raised by the Ministry of Labour and Social Inclusion and the employers associations about whether an accreditation system would lead to a better quality of OHS [3]. Most OHS, however, were in favour of an accreditation system [4–6].

In 2011, we asked all the Norwegian OHS about their experiences with the accreditation system. The response rate was 75%. Half of them had had to make various adjustments in order to be accredited. More than half of OHS believed that accreditation had improved the quality of their services [7]. In 2011, experience with the accreditation system was limited. The aim of this study was to update the findings from 2011.

## Methods

We sent a 23-item web-based questionnaire to all the Norwegian OHS accredited by the Labour Inspection Authority, asking about the type and size of the OHS, their experiences and the necessary adjustments they had had to make in order to become accredited and future challenges for OHS. Only one response was allowed from each OHS. We designed the questionnaire in cooperation with the OHS professional associations and the accreditation unit. We conducted two pilot studies before finalizing the questionnaire, which we sent out in November 2014. After three reminders, the survey was completed in January 2015.

In Norway, ethical approval is not necessary for this kind of study, since no health information was asked for.

## Results

We received responses from 76% (173) of the 228 accredited OHS, with 2400 employees (2000 FTEs) and serving 26000 companies with 1.1 million employees. The OHS were classified as external (68%), serving several enterprises, and internal (32%) serving only one enterprise.

Forty-two per cent of OHS reported that they had made various adjustments to achieve accreditation. The most commonly reported measures were improvements of the quality system (53%; 34 OHS), implementing a training programme for the OHS staff (44%), increased staffing in occupational hygiene (36%) and occupational medicine (28%) and partnering with another OHS (9%).

The OHS reported that the accreditation had led to better quality assurance (56%), better professional competence (42%), increased multidisciplinary working (38%), improved cooperation with the customer businesses (23%) and more networking with other OHS (22%). Fifty-six per cent reported that the accreditation system had led to a better overall quality of their own OHS and 47% to a better quality of OHS in Norway (Table 1).

**Table 1.** Impact of the accreditation on OHS self-reported quality

	Yes, *n* (%)	No, *n* (%)	Not sure, *n* (%)
Has the accreditation improved the quality in your OHS? (*n* = 156)	87 (56)	39 (23)	30 (17)
Has the accreditation improved the quality of OHS in Norway? (*n* = 155)	73 (47)	17 (11)	67 (42)

Half (51%) of the OHS reported that they were satisfied with the accreditation system (Table 2).

**Table 2.** How satisfied are you with the accreditation system (*n* = 158)?

	*n* (%)
Very satisfied	14 (9)
Satisfied	66 (42)
Neither satisfied nor dissatisfied	48 (30)
Dissatisfied	25 (16)
Very dissatisfied	5 (3)

We also asked the OHS about current and future challenges. Competition between OHS driving down prices (50%; 79), the lack of specialists in occupational medicine (26%), requests for ancillary services (general health check-ups, physical fitness, etc.) (16%) and small businesses being rejected by OHS for profitability reasons (16%) were some of the challenges mentioned.

## Discussion

The survey found that 56% of OHS reported that the accreditation system in Norway has led to a higher quality in their OHS and in OHS in Norway (47%). Improvement of quality systems, competence level and increased staffing in occupational hygiene and occupational medicine were the most common areas of improvement. They reported that this had led to better quality assurance of the services provided by the OHS, better expertise, greater multidisciplinarity and better cooperation with the businesses. Half of the OHS were satisfied with the accreditation system.

This study has some strengths and weaknesses. A major strength is the high response rate. Furthermore, the answers from the first mailing of questionnaires were very similar to those obtained from the three reminders. We therefore believe that the respondents were representative for OHS in Norway. We asked for OHS perception of quality. There are many other measures of quality [8]. In this study, assessment was based on self-report. We used a non-validated questionnaire, since there are few, if any, validated questionnaires on experiences with OHS accreditation. We designed the questions in collaboration with the OHS and the accreditation unit in the Labour Inspection Authority and piloted them twice on a small group of OHS. We believe this contributed to the quality of the questions.

The responses from OHS are similar with those in the 2011 study when the accreditation system was fairly new. Since then, the accreditation unit has inspected most of the OHS, and the accreditation system has been widely discussed. Support for the accreditation system is still strong. Other countries may have different experiences. When the Netherlands introduced a mandatory certification for its OHS some years ago, it was finally perceived as unnecessary, bureaucratic, costly and did not lead to better quality according to the OHS and their customers. The Dutch certification system was therefore abandoned [9]. The Norwegian accreditation unit guides OHS through the accreditation process and accreditation is free of charge. These may be some of the reasons why accreditation is better accepted in Norway than in the Netherlands.

**Key points**

The Norwegian occupational health service accreditation system introduced in 2010 is well accepted by accredited occupational health services.
Accredited occupational health services in Norway report that the accreditation process has improved the quality of their occupational health services and occupational health services in Norway.

## Conflicts of interest

None declared.
